# Evaluation of classifications of the monopodial bronchopulmonary vasculature using clustering methods

**DOI:** 10.1007/s00418-022-02116-x

**Published:** 2022-06-23

**Authors:** Jonas Labode, Christian Dullin, Willi L. Wagner, Despoina Myti, Rory E. Morty, Christian Mühlfeld

**Affiliations:** 1 Institute of Functional and Applied Anatomy, Hannover Medical School, Carl-Neuberg-Str. 1, 30625 Hannover, Germany; 2 Biomedical Research in Endstage and Obstructive Lung Disease Hannover (BREATH), Member of the German Center for Lung Research (DZL), Hannover, Germany; 3 Department for Diagnostic and Interventional Radiology, University Medical Center Göttingen, Robert-Koch-Str. 40, 37075 Göttingen, Germany; 4 Department of Diagnostic and Interventional Radiology (DIR), University of Heidelberg, Heidelberg, Germany; 5 Translational Lung Research Center (TLRC), Member of the German Center for Lung Research (DZL), University of Heidelberg, Heidelberg, Germany; 6 Department of Lung Development and Remodelling, Max Planck Institute for Heart and Lung Research, Bad Nauheim, Germany; 7 Department of Translational Pulmonology, University Hospital Heidelberg, Heidelberg, Germany

**Keywords:** Pulmonary vasculature, Monopodial lung, Branching analysis, Cluster analysis, Synchrotron micro-CT, 3D reconstruction

## Abstract

Mammalian pulmonary arteries divide multiple times before reaching the vast capillary network of the alveoli. Morphological analyses of the arterial branches can be challenging because more proximal branches are likely biologically distinct from more peripheral parts. Thus, it is useful to group the arterial branches into groups of coherent biology. While the generational approach of dichotomous branching is straightforward, the grouping of arterial branches in the asymmetrically branching monopodial lung is less clear. Several established classification methods return highly dissimilar groupings when employed on the same organ. Here, we established a workflow allowing the quantification of grouping results for the monopodial lung and tested various methods to group the branches of the arterial tree into coherent groups. A mouse lung was imaged by synchrotron x-ray microcomputed tomography, and the arteries were digitally segmented. The arterial tree was divided into its individual segments, morphological properties were assessed from corresponding light microscopic scans, and different grouping methods were employed, such as (fractal) generation or (Strahler) order. The results were ranked by the morphological similarity within and dissimilarity between the resulting groups. Additionally, a method from the mathematical field of cluster analysis was employed for creating a reference classification. In conclusion, there were significant differences in method performance. The Strahler order was significantly superior to the generation system commonly used to classify human lung structure. Furthermore, a clustering approach indicated more precise ways to classify the monopodial lung vasculature exist.

## Introduction

In a recent study (Grothausmann et al. 2021), a fractal branching pattern based on the definition by Wang and Kraman (2004) was used to classify the pulmonary artery branches of a rabbit lung. The resulting groups of arterial generations were subjected to stereological estimation of arterial wall characteristics. A high morphological dissimilarity within the resulting groups was observed, suggesting that the groups consisted of biologically non-coherent arterial branches. This prompted the search for a better grouping method for the monopodial lung.

A classification of the segments of the vascular tree into homogeneous groups is an invaluable tool to identify and compare morphologically and functionally similar structures, e.g., between healthy and pathological tissue. Potentially, this allows a detailed characterization of developmental or pathophysiological processes by gaining site-specific knowledge about the preferential localization of on-going processes. In the lung, this might help extend the current methods for the study of the pulmonary valculature (see, e.g.,Mühlfeld 2021) to further analyze the effects and development, e.g., of cystic fibrosis, bronchopulmonary dysplasia or chronic obstructive pulmonary disease.

The monopodial lung architecture is characterized by an asymmetrical branching pattern, centered around a main trunk stretching through the complete length of the lobe. Smaller vessels branch off the central trunk in an approximately perpendicular fashion. This branching pattern is often found in smaller mammals, e.g., mice, rats and rabbits, but also dogs and pigs (see, e.g., Schittny 2017, Monteiro and Smith 2014) and differs greatly from the dichotomous branching of the human lung, in which vessels usually split into two roughly symmetrical daughter branches; see, e.g., Weibel (1963).

Multiple grouping algorithms for the classification of lung vasculature can be found in the literature. Commonly known examples are generations (Ochs and Weibel 2008) which are a widely used metric to classify human airway and pulmonary artery branching, orders (Horsfield 1984), Strahler order (Strahler 1957) or the aforementioned fractal branching (Wang and Kraman 2004) as a method aimed especially at the classification of monopodial lungs.

These algorithms are usually established by proving a (often logarithmic) relationship between a group designation and the mean dimension of one or multiple morphological features (e.g., diameter or length) (see Wang and Kraman 2004 or Horsfield 1978). Other studies employed one method (e.g., orders) and used the results to characterize branching differences between species (e.g., Phillips and Kaye 1995). Comparisons between different methods are rare and only in discussion form, where the suitability of a method for a certain task is deemed unfit, because a type of distinct vessel is sorted into a wide variety of generations (see, e.g., Singhal et al. 1973).

Given the different geometric assumptions on which the classifications are based, the resulting groups will show different levels of internal morphometric similarity and will differ in number and size. This raises the question of which of the above-mentioned methods is suited best for the monopodial lung and whether there are even other ways to classify the pulmonary artery branches which might be superior to the currently known methods.

Thus, the aim of this study was to establish a workflow to measure the quality of vessel groupings and thereby create a basis for the selection of a suited algorithm as well as a way to evaluate new grouping algorithms possibly better suited for the monopodial lung architecture than previous methods.

Fundamentally, the classification of lung vasculature belongs to the domains of cluster analysis (see, e.g., Hastie et al. 2009) and unsupervised learning, where no reference solution is known to verify the performance of a particular algorithm. Instead, the underlying data structure needs to be uncovered without any external support (see, e.g., Hertz et al. 1991).

**Fig. 1:**
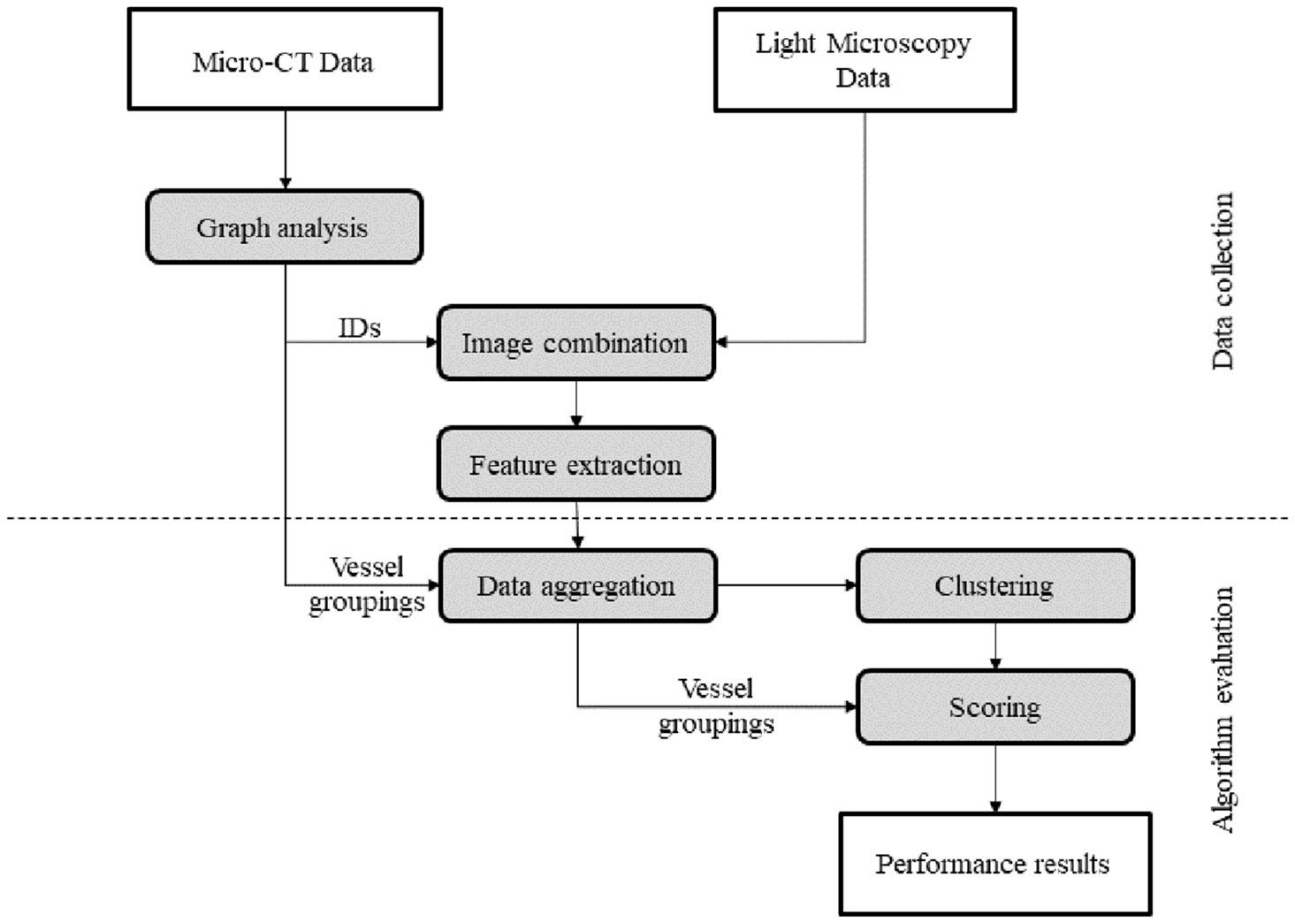
Schematic of the proposed workflow

The workflow presented in this paper (Fig. 1) consists of a phase of data collection (including imaging by $\upmu$ CT and light microscopy, integration of the source data and morphometry of individual arterial segments) and of an evaluation phase which forms the core of the present study. During this phase, the morphometric data are used to classify the different arterial branches according to the different algorithms and evaluate the quality of the groupings by methods of cluster analysis.

## Materials and methods

### Mouse lung processing

The lung used for this study was taken from a set of experiments for a previous study on bronchopulmonary dysplasia (see Appuhn et al. 2021). The study was approved by the Regierungspräsidium Darmstadt (approval no. B2/277). In short, newborn mouse pups were exposed to 85% $O_{2}$ for 14 days (Nardiello et al. 2017) and subsequently killed by intraperitoneal administration of sodium pentobarbital (Boehringer). The lungs were then fixed by vascular perfusion via the right ventricle at an inflation pressure of 20 cm$H_2O$ and a perfusion pressure of 20 cm$H_2O$. The fixative contained 1.5% (m/v) paraformaldehyde (Sigma) and 1.5% (m/v) glutaraldehyde (Serva) in 150 mM HEPES (Sigma). The whole left lung was then subsequently incubated with 1% (m/v) osmium tetroxide (Roth) in 0.1 M sodium cacodylate (Serva), 2.5% (m/v) uranyl acetate (Serva) in dd$H_2O$ and ascending acetone concentrations before embedding in glycol methacrylate (Technovit 8100; Heareus Kulzer).

### Synchrotron imaging

The embedded mouse lung was scanned at the SYRMEP beamline (SYnchrotron Radiation for MEdical Physics) of the Italian synchrotron “Elettra” using the “white beam” setup with the following parameters: 1800 projection images over 180$^{\circ }$, pixel size 4.4 $\upmu$m, sample-to-detector distance 450 mm and 1-mm silica filter resulting in an average x-ray photon energy of 19 keV (Dullin et al. 2021).

**Fig. 2:**
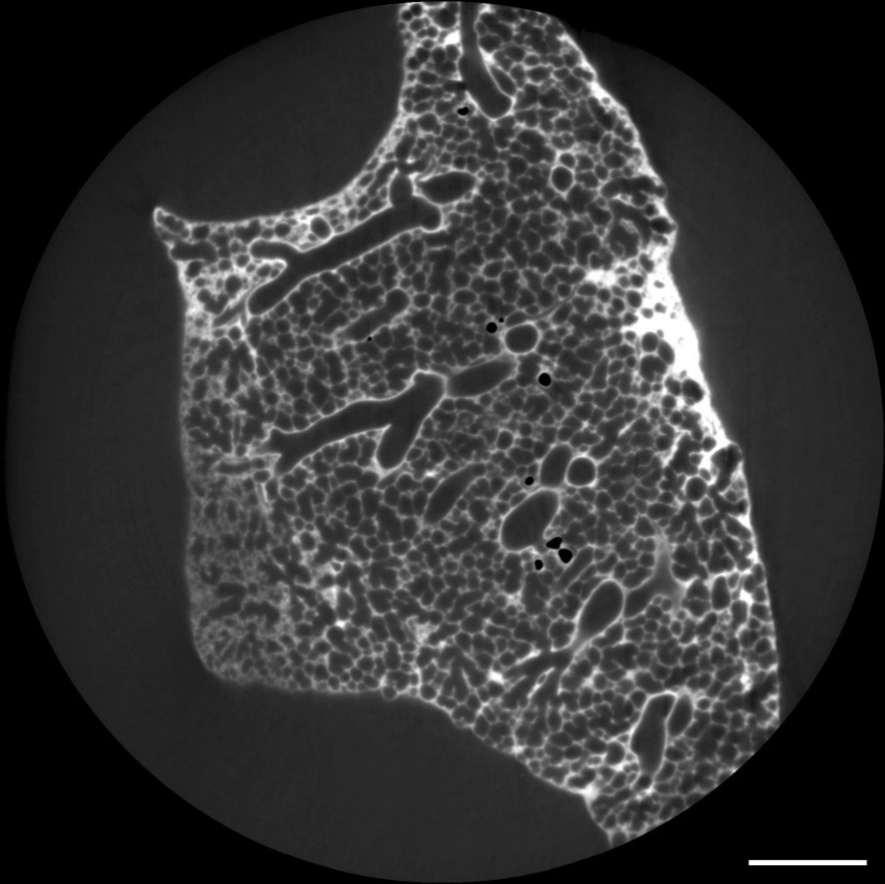
Synchrotron image of the mouse lung used for segmentation. Scale bar = 1 mm

The TIE_HOM phase retrieval algorithm (Paganin et al. 2002) for free propagation phase contrast imaging was applied with a delta-to-beta ratio of 100 prior to 3D reconstruction using filtered back projection, both implemented in the STP reconstruction program (Brun et al. 2015).

The imaging process could provide a volume image with a size of 1700 × 1700 × 1400 voxels (7480 × 7480 × 6160 $\upmu$m). Figure 2 shows a z-slice of this volume. The circular imaging area with a diameter of 7.48 mm was too small to fit the whole sample lung. This caused the three most distal vessel segments to be cut off prematurely. A comparative situation is present at the hilus-facing end in that the vessel tree starts just before its first bifurcation. Therefore, the imaging process dictates the definition of the start of the vessel tree.

### Lung sectioning

After synchrotron imaging, the complete lung was cut into a series of 2-$\upmu$m-thick slices, which were then mounted on glass slides and stained with toluidine blue. A total of 1401 slices were cut with only ten being directly excluded because of severe cutting artifacts.

Light microscopic imaging was conducted with a Zeiss Axioscan (Zeiss, Göttingen, Germany) slide scanner at 10× magnification.

### Segmentation and identifier attribution

The $\upmu$ CT data set was segmented to create a three-dimensional model of the arterial tree, which was then reduced to a 2D graph for analysis (Grothausmann et al. 2021). Deviating from the previous approach, this graph was further analyzed using a custom Python v3.7 (https://www.python.org/) program (https://github.com/labode/genana_py, commit e3cbafc), employing the NetworkX graph library v2.2 (Hagberg et al. 2008). The program was used to attribute a unique identifier number to every edge in the graph. This attribution was exported and mapped back into the initial 3D volume segmentation of the vessel tree. Further analysis of the 2D graph will be described in detail in the branching analysis section.

All steps in this software pipeline (including the ones described in the following sections) were organized in Makefiles (https://www.gnu.org/software/make/, v5.2.1) to manage dependencies and create a reproducible workflow. GNU parallel v20161222 (Tange 2011) was employed for parallel processing. Tracking of the development was done using git v2.20.1 (https://gitscm.com/) and git-annex v7.20190129 (https://git-annex.branchable.com/).

### Combination of $\upmu$CT and LM images

The LM images of the tissue were registered to one another to turn them from 2D images into a 3D volume image using CZIto3D (https://github.com/romangrothausmann/CZIto3D.git, commit 4dafe551f1) and the python script recRegStack.py (https://github.com/romangrothausmann/elastix_scripts.git, commit 82af09f), which employs SimpleElastix (Marstal et al. 2016) for the registration. Deviating from Grothausmann et al. (2021), an entire lung was used instead of only sample sections. Due to missing and unusable slices, there was still a need to generate multiple volumes of consecutive sections along the z-axis. In the gaps between these volumes, no composite images could be generated. This resulted in slight variations in the step size between two measurements, which are quantified in the following section.

Of the 1391 slices scanned, 81 were excluded because they contained too many cutting artifacts, thus disabling their use in the registration. The set of images was divided into 62 stacks of consecutive images. The first and last six stacks (281 sections) had too little material for a decent intra-stack image registration or did not include vessel markings and were therefore excluded from the further analysis. The $\upmu$ CT volume image was registered to these volumes to compensate for morphological deviations introduced by the cutting process. By using the resulting registration parameters on the segmentation image labeled with the IDs as described in the previous section, the LM volume images could be overlaid with those ID labels. The combined 3D image volumes where then again separated along their z-axis into 2-$\upmu$m-thick 2D images (*n* = 1029) for the following analysis. To aid the interpretation of the images, text labels were also added using another custom Python program (https://github.com/labode/label_image_py, commit 3245608, employing https://github.com/python-pillow/Pillow v.5.4.1).

### Morphometric feature extraction

To further characterize the arterial segments, vessel diameter and wall thickness were estimated using Fijis (Schindelin et al. 2012) line-drawing tool on the generated images to measure inner and outer vessel diameter. On every tenth of the 1029 images, the measurement lines were drawn perpendicular to the longitudinal axis in the center of each vessel segment with an ID label. This equals a regular step size of 20 $\upmu$m. In some cases, the steps were larger, where images between two stacks had to be excluded. This affected a section in the center of the z-axis, with a range of ten consecutive slices (= 20 $\upmu$m) missing and seven additional gaps distributed over the length of the entire axis, with two to three slices missing each.

The reduction of the data by 90% was performed to keep the manual work to a reasonable amount. Of the 102 images selected for analysis, 6 had to be excluded entirely because of severe misalignment between vessel segmentation and LM image. Several other images were affected by misaligned vessel profiles in a small extent, often affecting only some small peripheral vessel segments. The results of the measurements taken for each image were combined and averaged (if a segment was present in multiple images), using a simple Python program (https://github.com/labode/csv_merger_py, commit 64e3460). The wall thickness of each segment was calculated by subtracting its inner diameter from the corresponding outer diameter and multiplying the result by 0.5.

### Branching analysis

The branching classification methods were implemented using two different tools. The algorithms of generations, order and Strahler order were implemented in the Python program already employed for the ID attribution. It created a csv file output containing the ID of each vessel and the corresponding branching classifications. Additionally, the attributions were mapped back into the 3D vessel tree as described above. The fractal generations were modeled using the SGEXT (https://github.com/phcerdan/SGEXT, commit 141fde7) analysis module for an initial classification and manual overrides as necessary. They too were mapped back into the vessel tree for visualization.

All resulting files, sharing the vessel ID as a common identifier, could then be joined together for further analysis using the statistical computing environment R (R Core Team 2018).

### Grouping evaluation

A Gaussian mixture model (GMM, see, e.g., Fraley and Raftery 2007), as implemented in the R package mclust (Scrucca et al. 2016, v5.4.7), was used for clustering. It assumes that each cluster is composed of sample values distributed in a Gaussian fashion.

A limitation of GMMs is the fact that the number of clusters (denoted *k*) needs to be set before performing the calculations. This implies that the result will be a locally optimal cluster attribution for the defined number of clusters. Thus, a GMM on its own is not able to find the optimal number of clusters in a data set.

Therefore, the Bayesian information criterion (see Schwarz 1978) was used in this workflow for cluster counts from one to nine to estimate the optimal number of clusters in the data set, again using the R library mclust. Note that alternative metrics, such as the integrated complete-data likelihood, could be employed for this purpose as well (see Biernacki et al. 2000).

To compare the performance of the different clustering algorithms to be evaluated in this study, the R implementation (Walesiak and Dudek 2020, v0.49.2) of the Davies-Bouldin index (Davies and Bouldin 1979) was used. It generates an overall score that characterizes the data division achieved by each method.

## Results and discussion

### Morphometric data acquisition

**Fig. 3:**
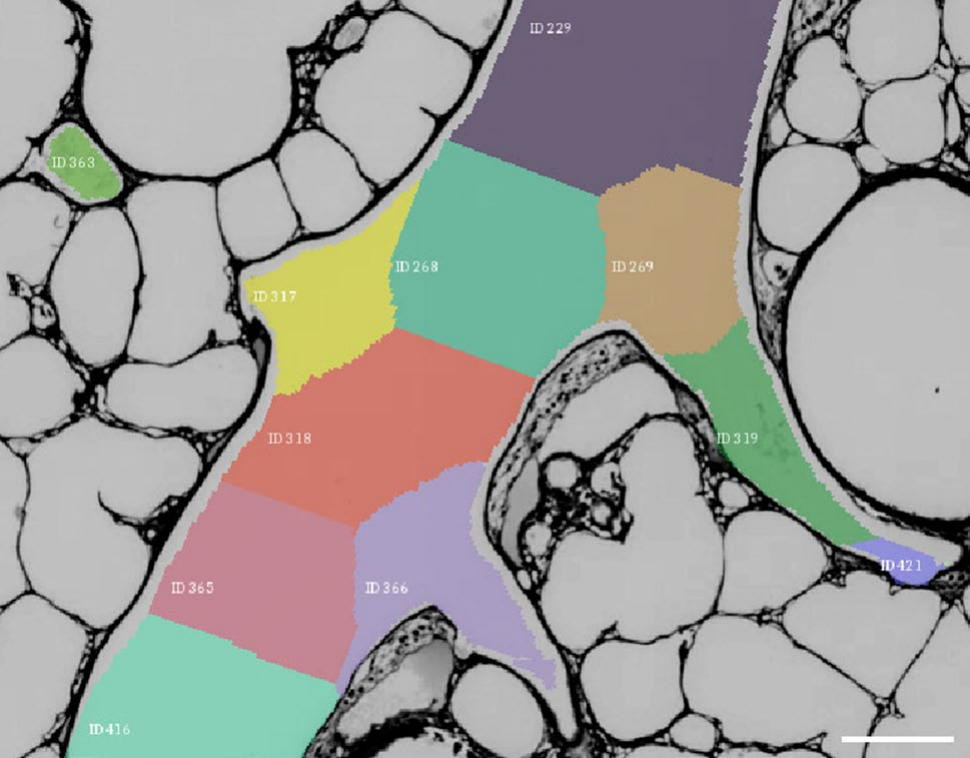
LM image of a mouse lung section. The IDs generated by dividing the arterial vessel graph into its segments between every bifurcation are marked with a both a color and a text label. Scale bar = 150 $\upmu$m

The workflow described by Grothausmann et al. (2021) was successfully adapted and extended to segment a 3D model of a vessel tree into its nuclear components, then they were labeled and mapped into 2D LM images. The referencing of every tree segment by a unique ID proved to be an important factor in aggregating both measurements and classification results. A subsection of an image prepared in this way can be seen in Fig. 3.

**Fig. 4:**
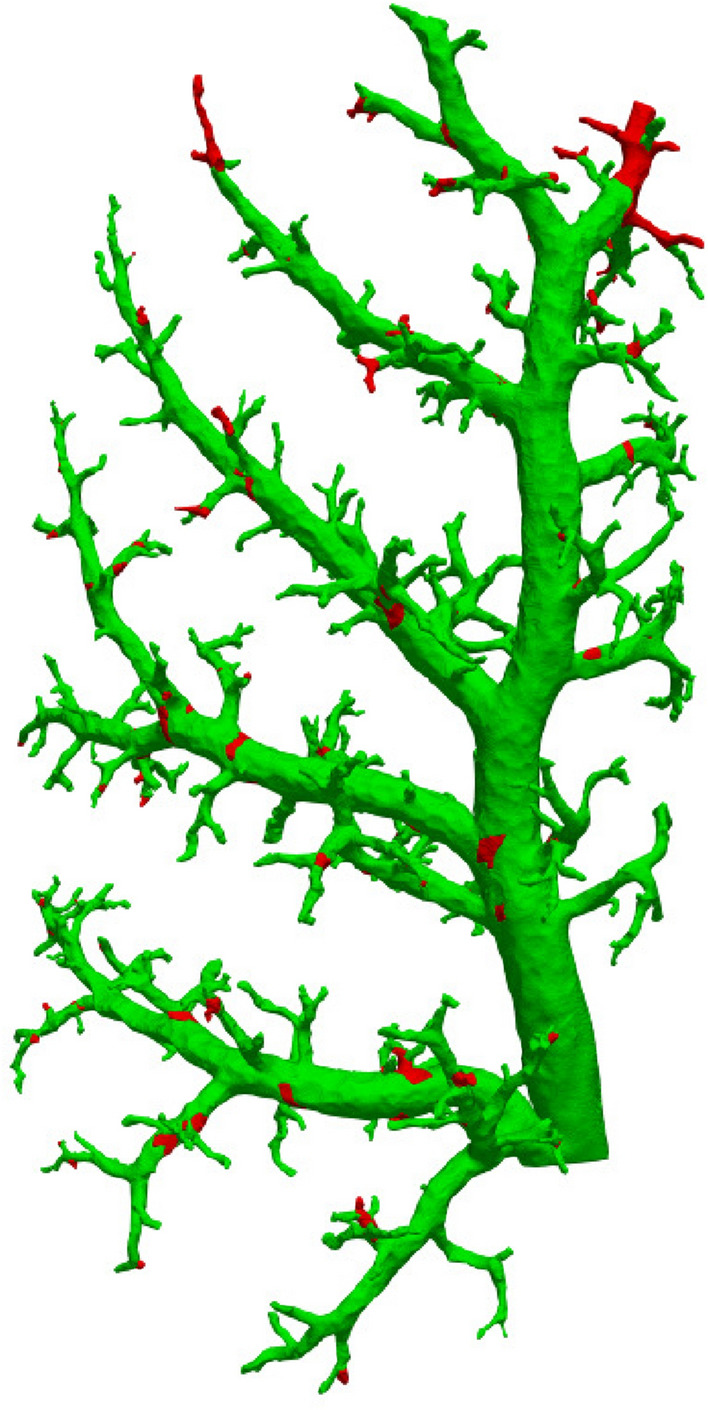
Data availability mapped to vessel tree. Segments for which measurements could be acquired are colored green; red sections mark unmeasured areas

In total, 883 edges (= distinct vessel segments) were identified in the graph analysis. Due to the data reduction, only 702 of the 883 segments could be measured in the combination images (79.5%). Overall, 2869 measurements were taken for inner and outer vessel diameter each, averaging to about four measurements per vessel segment. Figure 4 shows a rendering of the vessel tree. The vessel segments for which measurements were acquired are marked in green, whereas those segments that could not be measured because of misalignment or absence are marked in red. The visualization was created using the Python program https://github.com/labode/nrrd_translator_py (commit c5d2471), employing https://github.com/mhe/pynrrd v0.4.2. Rendering was done in Paraview v5.8.0 (Ahrens et al. 2005).

The limiting factor in the segmentation process was the $\upmu$CT image resolution of 4.4 $\upmu$m. In practice, the smallest vessels that could be identified in this resolution were small arterioles with about 30 $\upmu$m diameter. These would then branch off into the smaller precapillary vessels, leading to the capillary network where vessel diameters range between 5 $\upmu$m and 8 $\upmu$m (see, e.g., Townsley (2011)). In the currently used data set, blood vessels of < 30 $\upmu$m diameter were beyond the resolution limit of the imaging process.

### Lung branching classification

In addition to the ID attribution, multiple algorithms for lung branching classifications were implemented as a software tool.

**Fig. 5:**
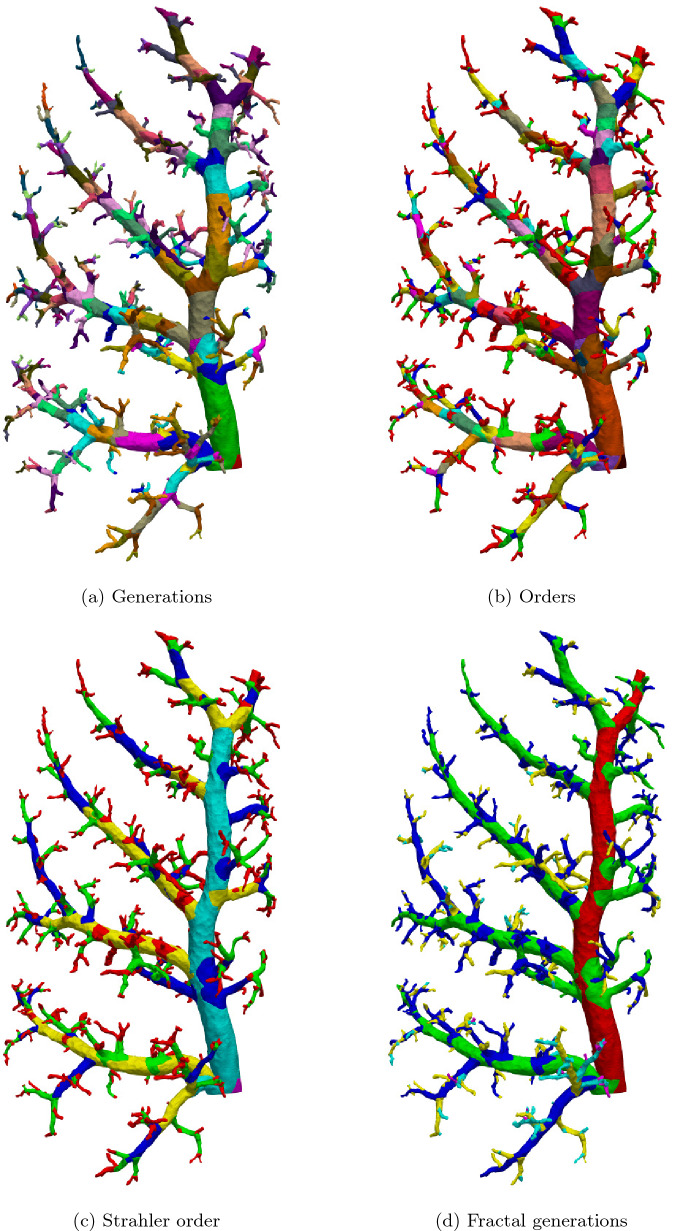
Different classification schemes for vessel segments. The color markings each represent one class

Below is a brief description of each of the used classification algorithms and their results for the sample lung. Renderings of the results can be seen in Fig. 5. For a more in depth description of each method, please refer to the corresponding literature referenced in the introduction. For the **generational** approach (Fig. 5a), the graph edge emerging from the root node (point closest to the trachea) was attributed generation 1. At every bifurcation of the graph, the previous generation incremented by one was attributed to the daughter branches. This classification resulted in 27 distinct vessel groups. The **order** (Fig. 5b) attribution inverts this process. The algorithm starts at the leaf nodes (terminal vessels closest to the capillary bed) of the graph, classifying the edges terminating there as order 1. Traveling up the graph in the direction of the root node, every time two or more branches meet, the highest order of the merging branches, incremented by one, is attributed to the new segment. Just as with the generation method, the result are 27 vessel groups. The **Strahler order** (Fig. 5c) can be seen as an adaption of the regular order. It starts and moves the same way as the order classification, with the difference that an order increase only occurs when two or more branches of equal order meet. If branches of unequal size merge, the continuing branch will be attributed the highest order number of the merged branches. This method resulted in six groups of arteries with the first group being identical to the one of the regular order algorithm. Finally, the **fractal generations** approach (Fig. 5d) identifies the main vessel trunk centered in the lung lobe from start to end as generation 1. The segments branching off it in an approximately perpendicular fashion are assigned generation 2 from branching point to tip. Segments branching off generation 2 are assigned generation 3 up to the tip and so forth. This process resulted in seven groups.

The algorithms used here are only a selection of a number of proposed algorithms found in the literature. Further examples include, e.g., the diameter-defined Strahler system (Jiang et al. 1994) or an order model for asymmetrical lung architectures defined by Horsfield et al. (1982). These could be integrated into the proposed workflow in the same manner.

**Fig. 6:**
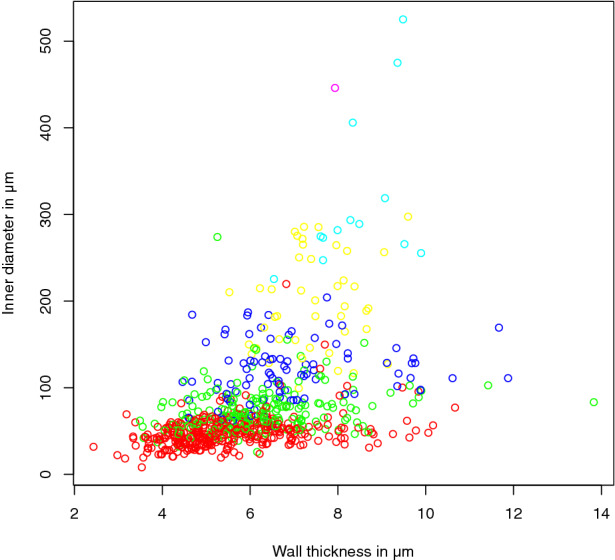
Plot of the vessel measurements colored according to their attributed Strahler order

When combining the group attribution and the vessel measurements, the spread of the morphometric features within those groups can be visualized by plotting these values. As an example, see Fig. 6 showing the Strahler order.

### Ranking of results

**Fig. 7:**
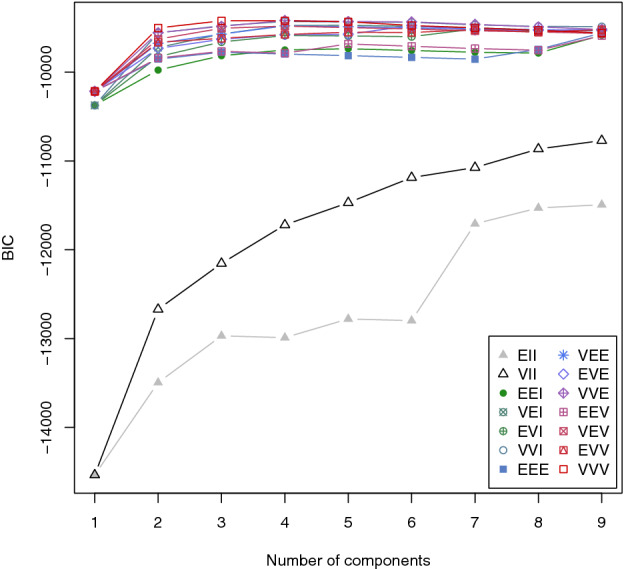
Bayesian information criterion for k1-k9

As the GMM method only provides a cluster solution for a given number of clusters, the optimal number of clusters in the data set had to be determined externally. This was done by calculating the Bayesian information criterion for the cluster counts 1 to 9 and 14 different geometric constellations. As shown in Fig. 7, a maximum Bayesian information criterion value (-9418.254) can be reached for four clusters in VVE geometry, an ellipsoidal distribution with variable volume and variable shape, but equal orientation (see Scrucca et al. 2016 for further information). Thus, the number of clusters for the GMM clustering was set to four.

**Fig. 8:**
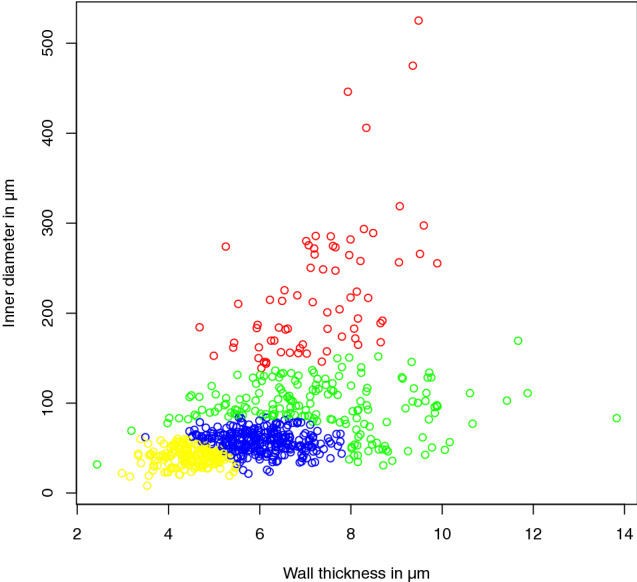
Plot of vessel measurements colored according to GMM results for four clusers

With this information on the optimal cluster count, the cluster membership for the data points was calculated using a GMM (Fig. 8).

There are multiple other clustering methods that could be employed in this workflow as well, for example, hierarchical clustering schemes as described, e.g., in Murtagh and Legendre (2014) or K-means clustering (e.g., Hartigan and Wong 1979).

**Fig. 9:**
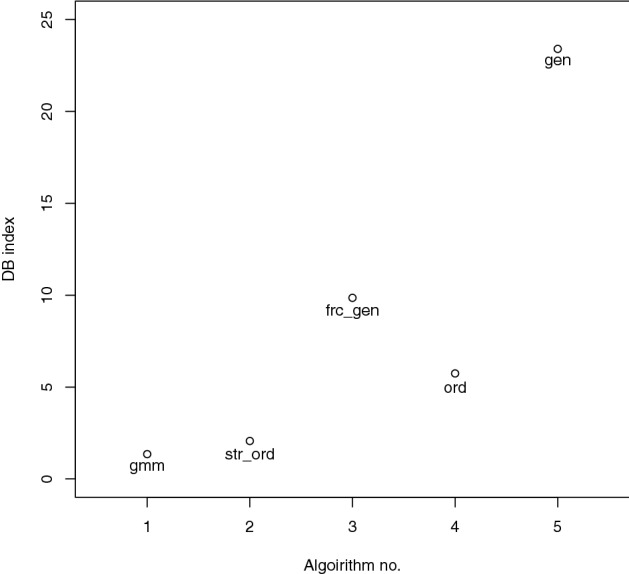
Davies-Bouldin index for all clustering results

Figure 9 shows the results of the Davies-Bouldin index for the calculated GMM clusters as well as the grouping algorithms based on graph locations. As the goal of clustering is to create distinct clusters of values, i.e., high heterogeneity between groups and the Davies Bouldin index calculates the average similarity between each cluster and its closest neighbor, a minimal Davies-Bouldin index identifies a well-clustered data set. GMM achieved a score of 1.35. This serves as a base measurement of how well the data set can be divided into clusters.

Among the evaluated algorithms, the Strahler order yielded the best score of 2.06, located nearest to the reference score. The other algorithms received much higher scores between 5.74 (order) and 23.40 (generations), revealing their limited accuracy in classifying the arterial branches.

Importantly, the order algorithm produced multiple clusters that contained only a single data point or were entirely empty (due to the single point in the cluster being one of the vessels not measured, see section morphometric feature extraction). This positively affected its Davies-Bouldin index, without adding any meaningful data separation. Generally, clusters without scores are a limitation of the employed data collection and evaluation methods and need to be taken into account when interpreting the results. Other potential methods that might be suited to be applied in this workflow include, e.g., the Dunn index (Dunn 1973, Brock et al. 2008).

**Table 1:** Davies-Bouldin index

**Algorithm**	**Score**
GMM	1.35
Strahler order	2.06
Fractal generations	9.85
Orders	5.74
Generations	23.40

The Davies-Bouldin index represents the final result of the presented workflow and helps identifying the best grouping algorithm of the arterial tree in the given lung. The Strahler order was the biologically defined grouping method with the best Davies-Bouldin index (Table 1), whereas the generations approach as a standard classification metric of dichotomously branching lungs performed worst. Despite the better results of orders and fractal generations, their use for monopodial lungs cannot be recommended, as their clusters were either not represented by the data structure or did not achieve a good separation between them, respectively.

### Qualitative discussion of clustering results

**Fig. 10:**
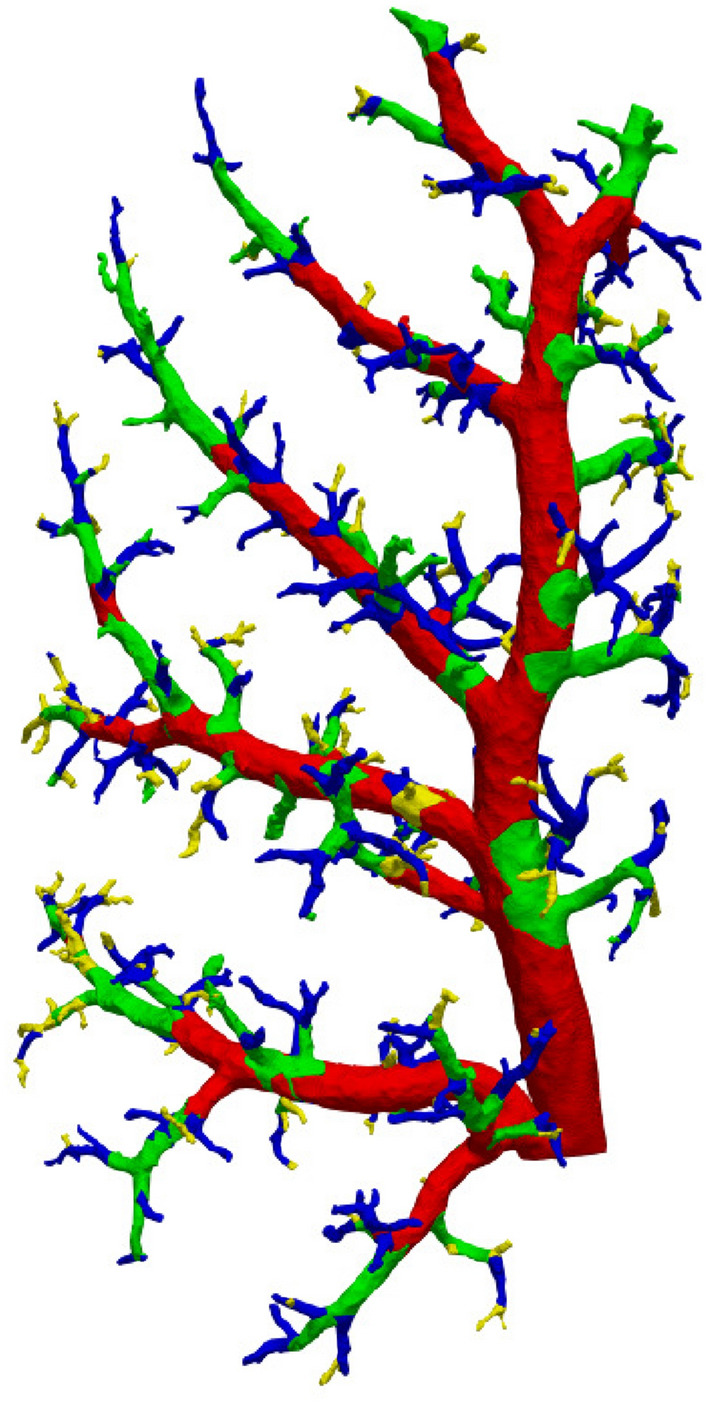
GMM clustering results mapped to graph. Vessel segments without measurements are labeled in equal parts with the labels of their bordering neighbor vessels

The Strahler order correlated well with the vessel diameter while seemingly having little correlation with the wall thickness (see Fig. 6). The GMM clustering scheme in combination with the Bayesian information criterion identified four distinct clusters and created an interesting classification, reducing the number of clusters and sharpening the borders between them and allowing an attribution to morphologically distinct vessel groups (Fig. 8 and 10, Table 2).

**Table 2:** GMM vessel grouping

**Color**	Approximate size ($\upmu$m)	**Characterization**
		**Large vessels**
Red	Diameter 150	Central branch segments
	**Diameter** **150**	**Small vessels**
Green	Diameter 75 or 4all 8	Non-terminal small vessels
Blue	Wall 6–8	Medium terminal vessels
Yellow	Wall 6	Small terminal vessels

### Parameter selection

For the mouse lung investigated in this study, the parameter wall thickness has to be regarded as a compromise. A more meaningful and informative measure would be the composition of the arterial wall structure, for example, the number of smooth muscle cell layers. However, the changes of the wall structure along the murine pulmonary arterial tree were only minimal, making a more precise distinction impossible (see, e.g., the wall structure in Fig. 3). Further studies, especially using larger mammalian lung samples, e.g., rabbit lungs, will probably provide a more detailed classification.

### Limitations of the workflow

Although the presented workflow provided a reasonable result, some limitations have to be mentioned. Of course, a more intuitive approach would be to use the light microscopic sections for both the segmentation process and the evaluation of the arterial morphology. This would avoid the use of the $\upmu$ CT imaging and the tedious registration of the $\upmu$ CT and LM data. Although this approach worked well in a small lung sample (Grothausmann et al. 2017), it is hardly possible for larger samples like a whole lung, even of a small species like the mouse. The unavoidable cutting artifacts in such a large series of thin sections create an accumulating registration error yielding an unusable 3D volume (see Grothausmann et al. 2021). The combination of $\upmu$ CT of the whole lung and subsequent serial sectioning combines the advantages of both methods while adding an acceptable level of extra work.

On the contrary, the information about vessel morphology that can be obtained from the $\upmu$ CT data is not sufficient to allow doing without the light microscopic imaging. Even with the use of synchrotron $\upmu$ CT imaging, the resolution of the microscope is currently superior, particularly with respect to the wall composition. Thus, the time-consuming light microscopic work still provides information that cannot be obtained by non-destructive imaging.

The efficiency of the proposed workflow is reduced by a high fraction of manual work that needs to be performed to achieve reasonable results. With respect to the segmentation process, advances in imaging technology as well as deep learning (e.g., Tan et al. 2021) will help to increase the possibility of full automation of the vessel tree segmentation.

## Conclusions

A workflow was presented to quantify the ability of different vessel classification schemes to group the vessel segments of a sample organ according to their morphological features. A data aggregation method integrating both $\upmu$CT and LM images was adapted for the generation of the required vessel measurements in a mouse lung. Additionally, a ranking method for the comparison of the results of different classification methods was employed. It was demonstrated that established clustering methods are able to provide better data separation than the algorithms usually used to classify lung vasculature. It will be necessary to evaluate whether the cluster pattern identified by the GMM algorithm is equally useful for other lungs.

Of the conventional classification methods, the Strahler order provided the best results for the present data set. Future studies need to explore further parameters (such as vessel wall composition) for the classification of the lung vasculature into biologically coherent groups.

## Data Availability

Image data are available upon request.

## Code availability

Makefiles and R-scripts are available upon request.
